# Supplementary material for: Modelling take-off moment arms in an ornithocheiraean pterosaur
Source: PeerJ. 2024 Aug 5;12:e17678. doi: 10.7717/peerj.17678 (PMC11308997; doi:10.7717/peerj.17678)

Institutional abbreviations

AMNH – American Museum of Natural History, New York, USA; BSP – Bayerische Staatssammlung für Paläontologie und Geologie, Munich, Germany; CMNH – Carnegie Museum, Pittsburgh, USA; SMNK – Staatliches Museum für Naturkunde, Karlsruhe, Germany; TMM – Texas Memorial Museum, Austin, USA; YPM – Yale University Peabody Museum of Natural History, New Haven, USA

Table S1:

Specimens examined for this study. Specimens were examined for articulation, morphology, and scarring caused by muscular and connective tissues to gain an understanding of the soft tissues across Pterosauria. Genus names used are based on the accession tags however several the specimens have subsequently been renamed in the literature.

| Genus | Specimen |
| --- | --- |
| *“Coloborhynchus”* | SMNK-PAL-1133, SMNK-PAL-1135, SMNK-PAL-1136 |
| *Rhamphorhynchus* | CM-11427, CM-11431, YPM-11984, YPM-1178, AMNH-FARB-1943,  AMNH-FARB-1943, AMNH-FARB-2031, AMNH-FARB-5135, BSP-1880-II-8, BSP-1925-I69, BSP-1964-XXIV-321, BSP-1971-I-68 |
| *Pteranodon* | YPM-VP-001164, YPM-1175, YPM-1181, YPM-2273, YPM-2344,  YPM-2347, YPM-2348, YPM-VP-002373, YPM-VP-002375, YPM-VP-002380, YPM-2414, YPM-2451, YPM-VP-002470, YPM-VP-002490, YPM-VP-002493, YPM-VP-002512, YPM-VP-002514, YPM -VP-002520, YPM-VP-002532, YPM-VP-002543, YPM-VP-002546, YPM-VP-002550, YPM-VP-002552, YPM-VP-002553, YPM-VP-002570, YPM-VP-002660, YPM-VP-002688, YPM-VP-002693, YPM-VP-002728, YPM-VP-002767, YPM-VP-002738, YPM-VP-002767, YPM-VP-002774, YPM-VP-002785, YPM-VP-002799, YPM-VP-042801, YPM-VP-042816, YPM-VP-042819, YPM-VP-042821, YPM-VP-062787, AMNH-FARB-2004, AMNH-FARB-2095,  AMNH-FARB-2122, AMNH-FARB-2123, AMNH-FARB-2171,  AMNH-FARB-2173, AMNH-FARB-2174, AMNH-FARB-2175,  AMNH-FARB-2176, AMNH-FARB-4903, AMNH-FARB-4906,  AMNH-FARB-4907, AMNH-FARB-4908, AMNH-FARB-5099,  AMNH-FARB-5819, AMNH-FARB-5840, AMNH-FARB-5841,  AMNH-FARB-7519, |
| *Nyctosaurus* | YPM-VP-001178, YPM-VP-002303, YPM-VP-002334, AMNH-FARB-143, AMNH-FARB-212 |
| *Anhanguera* | AMNH-FARB-22555, AMNH-FARB-22552 |
| *Brasileodactylus* | AMNH-FARB-24444, BSP-1991-I-27 |
| *Tapejara* | BSP-1992-I-62, SMNK-PAL-1137 |
| *“Araripesaurus”* | BSP-1982-I-90, BSP-1982-I-91, BSP-1990-I-45 |
| *“Santanadactylus”* | BSP-1980-I-120, BSP-1980-I-122, BSP-1982-I-89, BSP-1982-I-92,  BSP-1987-I-1, BSP-1893-I-92, BSP-1987-I-65 |
| *Arthurdactylus* | SMNK-PAL-1132 |
| *Quetzalcoatlus* | BSP-1978-I-[52-56], BSP-1992-I-[26-36] (replica of TMM 42180),  TMM-41544 cast stored at BSP |
| *Dimorphodon* | YPM-350 |
| *Dsungaripterus* | YPM-57204 |
| *Pterodaustro* | AMNH-FARB-22572, BSP-1975-I-23 |
| *Scaphognathus* | AMNH-FARB-1692, AMNH-FARB-1694 |
| *Dorygnathus* | AMNH-FARB-32179, SMNK (accession number unknown for casts) |
| *Pterodactylus* | AMNH-FARB-5134, BSP-1867-II-1, BSP-1875-XIV-501, BSP-1878-VI-1,  BSP-1936-I-50, BSP-1964-XXIII-100, BSP-1977-XIX-39, BSP-AS-I-739 |
| *“Aranpedactylus”* | BSP-1975-I-166 |
| *Gnathosaurus* | BSP-AS-VIII-369, BSP-1969-I-94 |
| *“Ornithocheirus”* | BSP-1986-I |
| *Germanodactylus* | BSP-1892-IV-1, BSP-1977-XIX-1, BSP-AS-I-745 |
| *“Gallodactylus”* | BSP (accession number missing but drawer named) |
| *Eudimorphodon* | BSP-1994-I-51 |
| *Anurognathus* | BSP-1922-I-42 |
| *“Rhamphocephalus”* | BSP-1976-I-[41-44] |
| *Arambourgiana* | SMNK-XLIV/2 |
| *Tupuxuara* | SMNK-PAL-6595 |
| *Lacusovagus* | SMNK-PAL-4325 |
| Tapejaridae, gen. indet. | SMNK-PAL-3843 |
| Pterodactyloidea, gen. indet. | AMNH-FARB-22553, AMNH-FARB-22569, AMNH-FARB-29422, |
| Ornithocheiroidea, gen. indet. | SMNK-PAL-1134, SMNK-PAL-1185, SMNK-PAL-2302, SMNK-PAL-3845, SMNK-PAL-3854 |
| Azhdarchoidea, gen. indet. | AMNH-FARB-22569, BSP-1976-I-64, SMNK-PAL-2342, SMNK-3842,  SMNK-PAL-3843, SMNK-3856, SMNK-PAL-3985, SMNK-PAL-3900 |
| Neoazhdarchia, gen. indet. | SMNK-PAL-9876 |
| Pterosauria, gen. indet | AMNH-FARB-22575, AMNH-FARB-23791, SMNK-PAL-2350,  SMNK-PAL-3830, SMNK-PAL-3984, SMNK-PAL-3986, SMNK-PAL-6607 |

Table S2: Muscle wrapping surfaces utilised in the musculoskeletal model. For cylinders the radius is shown in the radius(x) column

| Muscle | Location | Shape | r(x) | r(y) | r(z) | t(x) | t(y) | t(z) | Length | Rad(x) | Rad(y) | Rad(z) |
| --- | --- | --- | --- | --- | --- | --- | --- | --- | --- | --- | --- | --- |
| LD | Pectoral | Ellipsoid | -0.1 | 0 | 0 | 0.054 | -0.04 | 0.005 |  | 0.168 | 0.07 | 0.12 |
| DS,LD,SHA,TR-S | Pectoral | Cylinder | 2 | 1.4 | 1 | 0.022 | -0.027 | -0.121 | 0.06 | 0.019 |  |  |
| TR-C,TR-L,TR-M,TR-S | Humerus | Cylinder | 0.4 | 0.25 | 0 | 0.276 | -0.014 | 0.023 | 0.07 | 0.018 |  |  |
| FCU,FDL | RadUln | Cylinder | 4 | 0 | 0 | 0.36 | -0.022 | 0.072 | 0.04 | 0.01 |  |  |
| ECR | RadUln | Cylinder | 4 | 0 | 0 | 0.355 | 0.008 | 0.072 | 0.05 | 0.017 |  |  |
| FDB,FDL | WMC | Cylinder | 1.7 | 0.3 | 1.3 | 0.259 | 0.067 | 0.028 | 0.04 | 0.02 |  |  |
|  | Pelvis | Ellipsoid | 0 | 0 | 0 | -0.012 | 0 | 0 |  | 0.06 | 0.05 | 0.035 |
| IFM | Femur | Ellipsoid | 0 | 0 | 0 | 0 | 0 | 0 |  | 0.11 | 0.11 | 0.11 |
| FMTE,FMTI,ITB, | Femur | Cylinder | 4.7 | 0 | -0.3 | 0.268 | 0.006 | 0.001 | 0.028 | 0.012 |  |  |


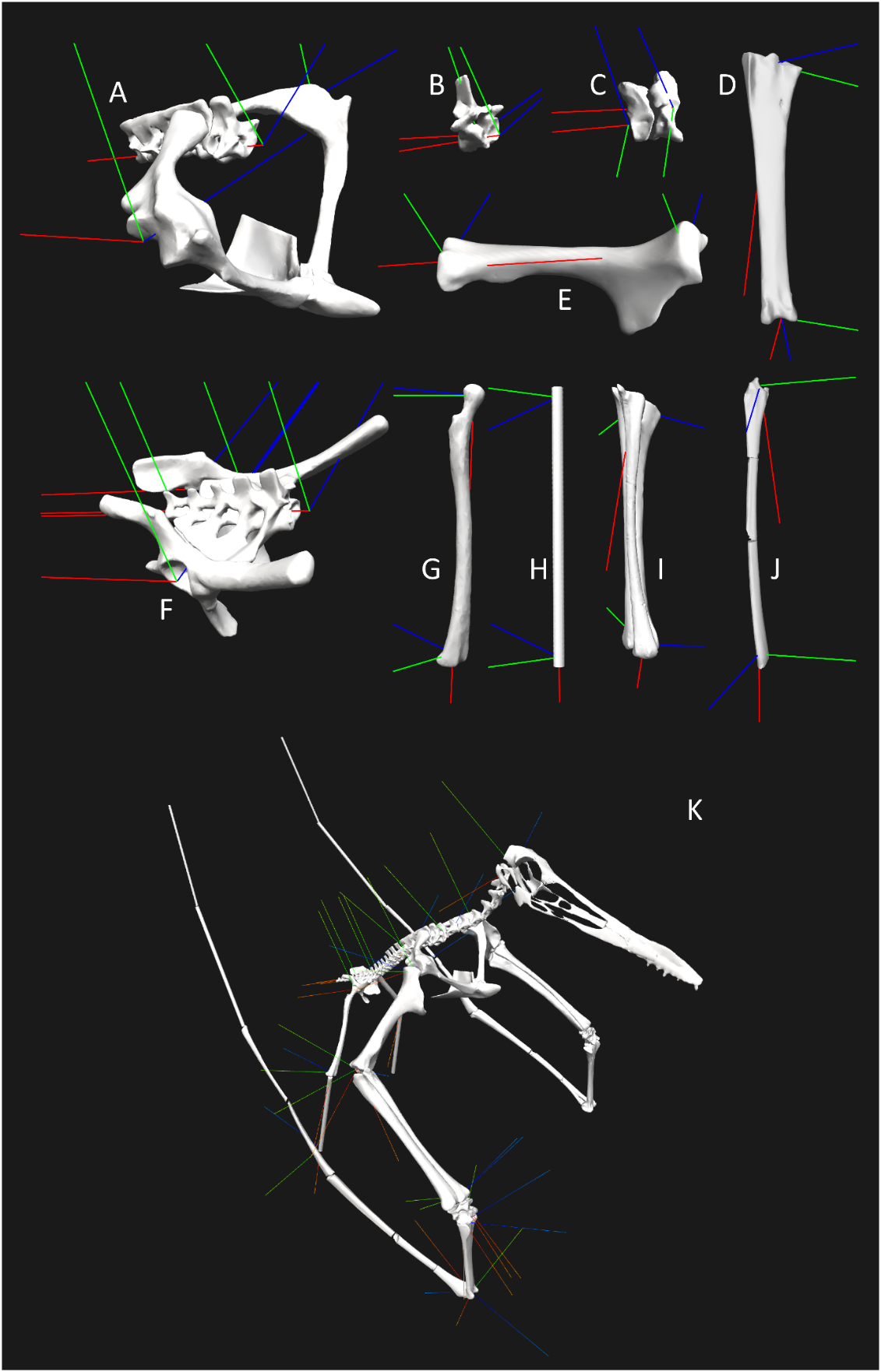
Figure S1: Model joint coordinate system. A) pectoral girdle, B) example vertebrae, C) combined right carpals, D) right wing metacarpal, E) right humerus, F) pelvic girdle, G) right femur, H) example scaled cylinder for missing element, I) combined right radius and ulna, J) right wing phalanx 1, K) complete skeletal model in quadrupedal stance with right side and representative axial joint coordinate system orientations.


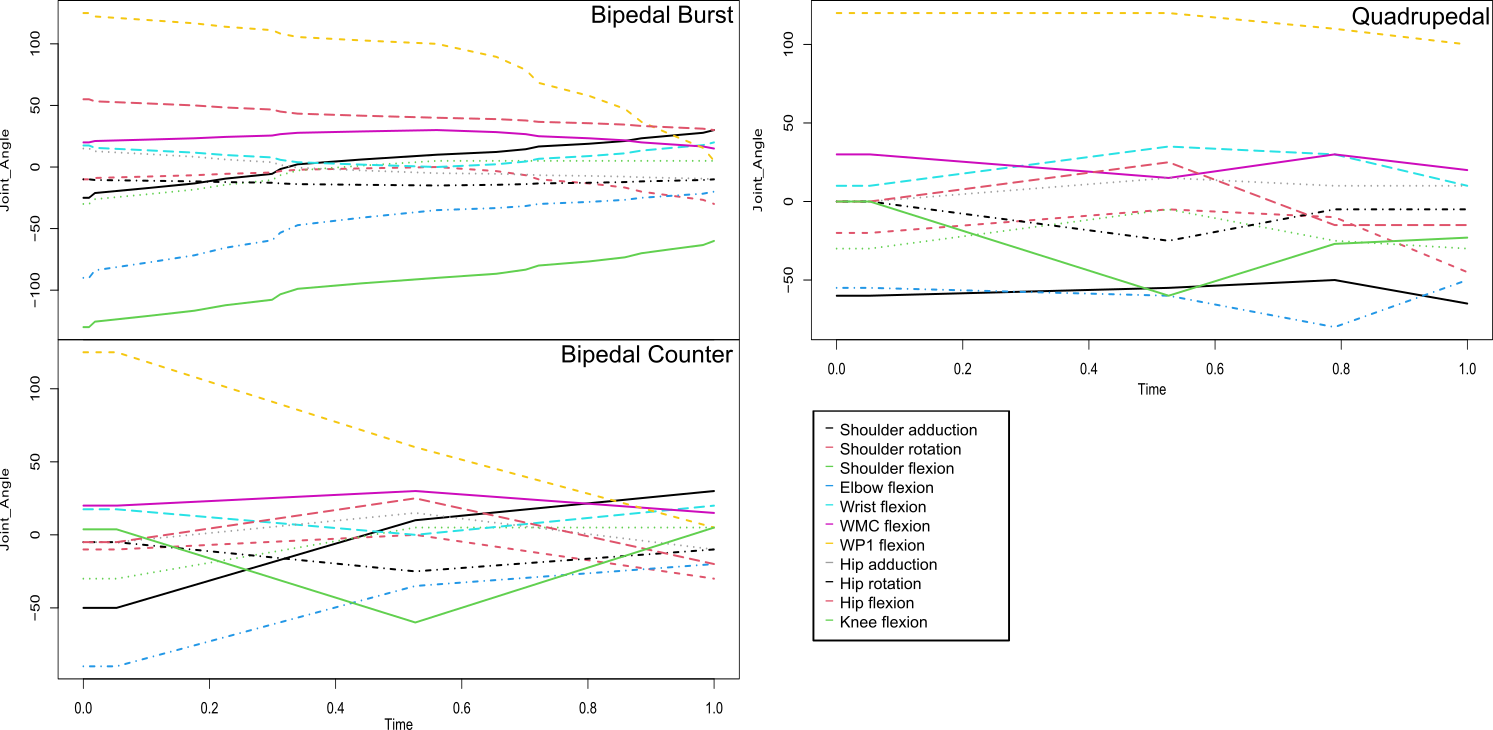
Figure S2: Joint angles relative to the OpenSim model zero pose through each of the take-off kinematics.


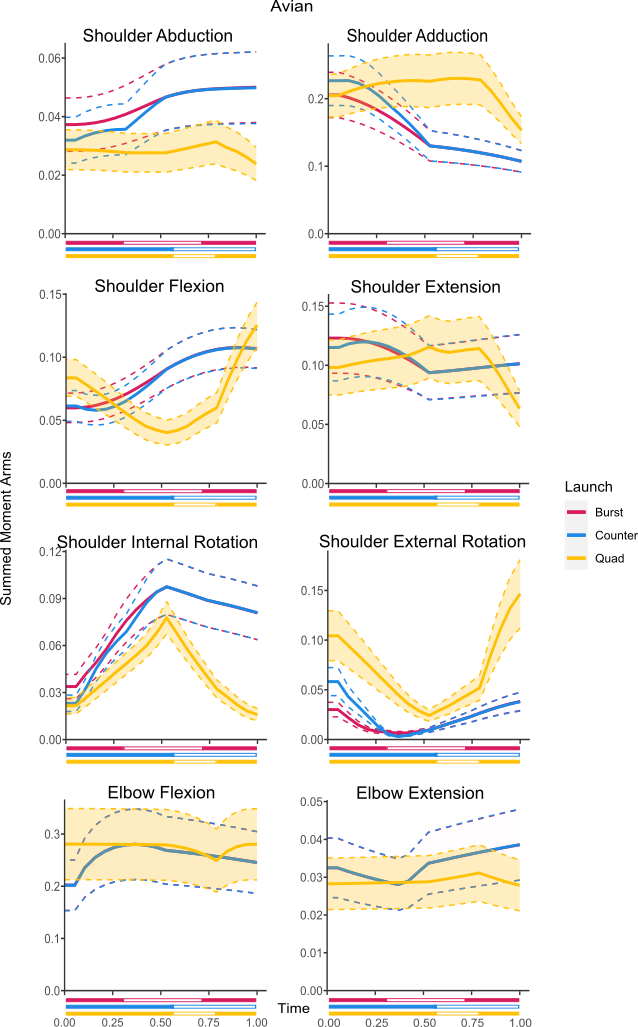


Figure S3: Summed moment arms in each hypothesised take-off motion for the lower forelimb rotational DOFs utilising only muscles present in avians. Solid lines indicate mean values following Monte Carlo simulation, dashed lines show estimated error, colouration indicates moment arm usage throughout the take-off.


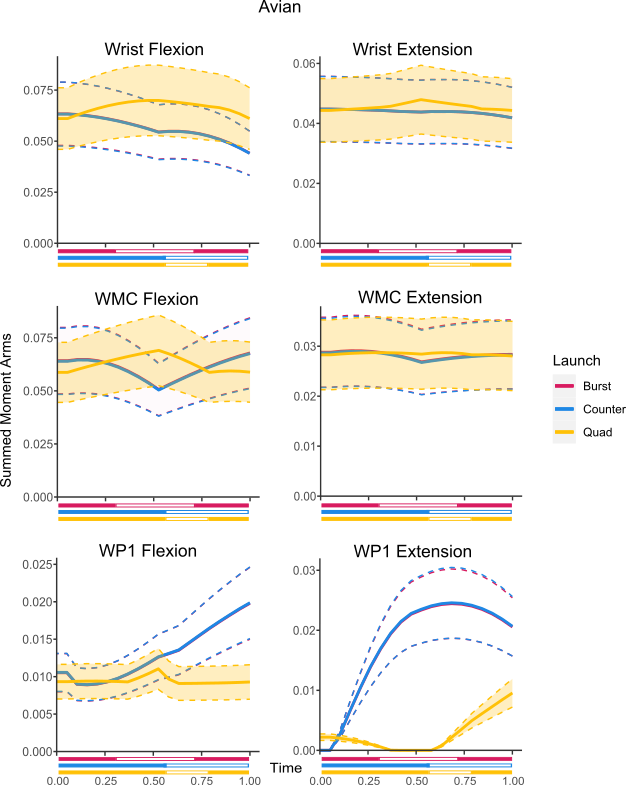


Figure S4: Summed moment arms in each hypothesised take-off motion for the hindlimb rotational DOFs utilising only muscles present in avians. Solid lines indicate mean values following Monte Carlo simulation, dashed lines show estimated error, colouration indicates moment arm usage throughout the take-off.


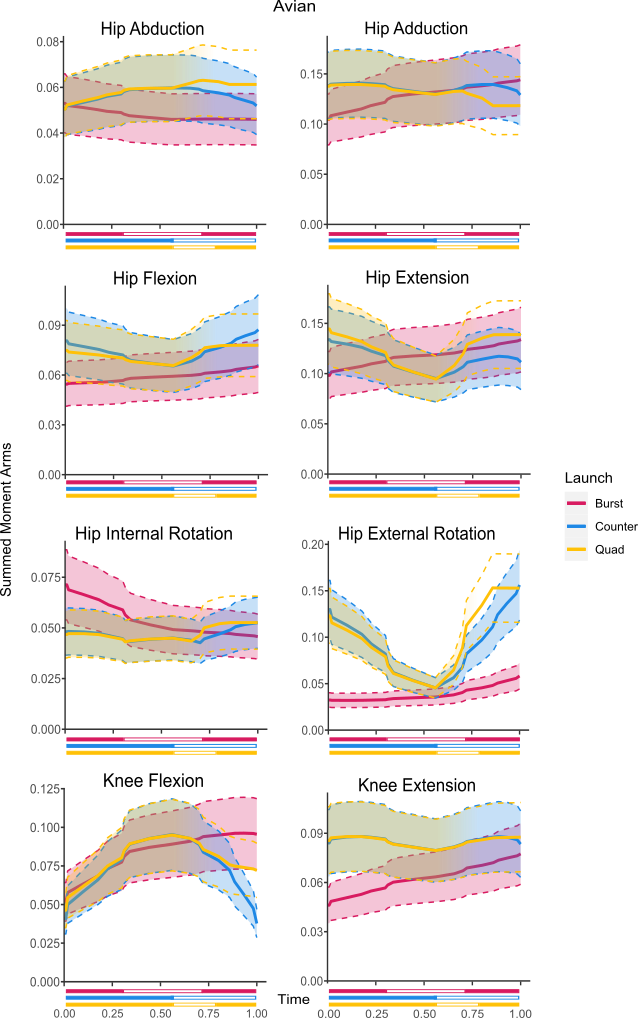


Figure S5: Summed moment arms in each hypothesised take-off motion for the upper forelimb rotational DOFs utilising only muscles present in crocodilians. Solid lines indicate mean values following Monte Carlo simulation, dashed lines show estimated error, colouration indicates moment arm usage throughout the take-off.


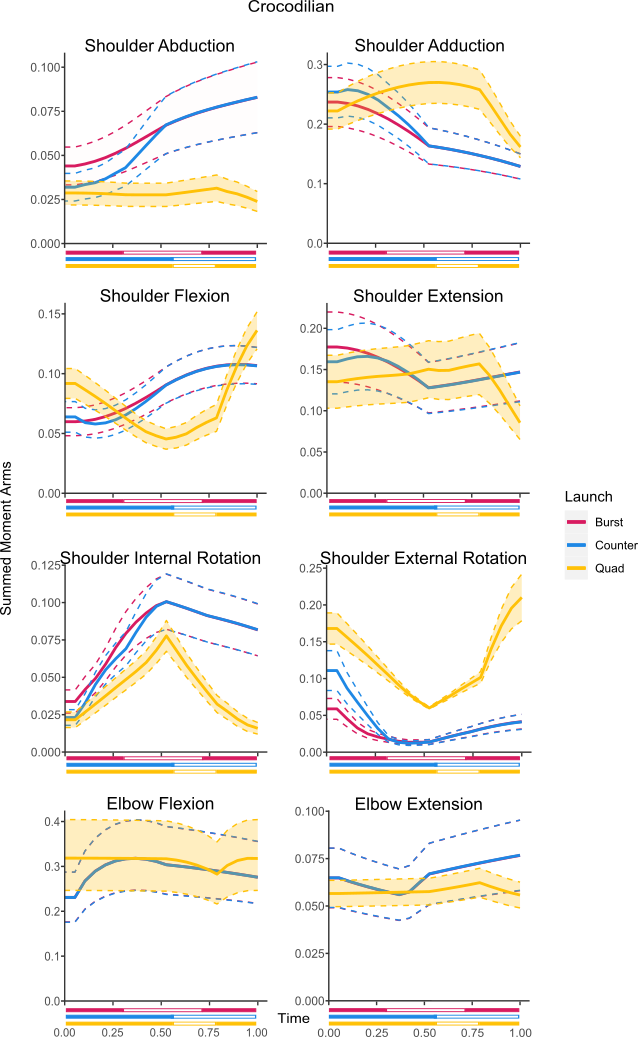


Figure S6: Summed moment arms in each hypothesised take-off motion for the lower forelimb rotational DOFs utilising only muscles present in crocodilians. Solid lines indicate mean values following Monte Carlo simulation, dashed lines show estimated error, colouration indicates moment arm usage throughout the take-off.


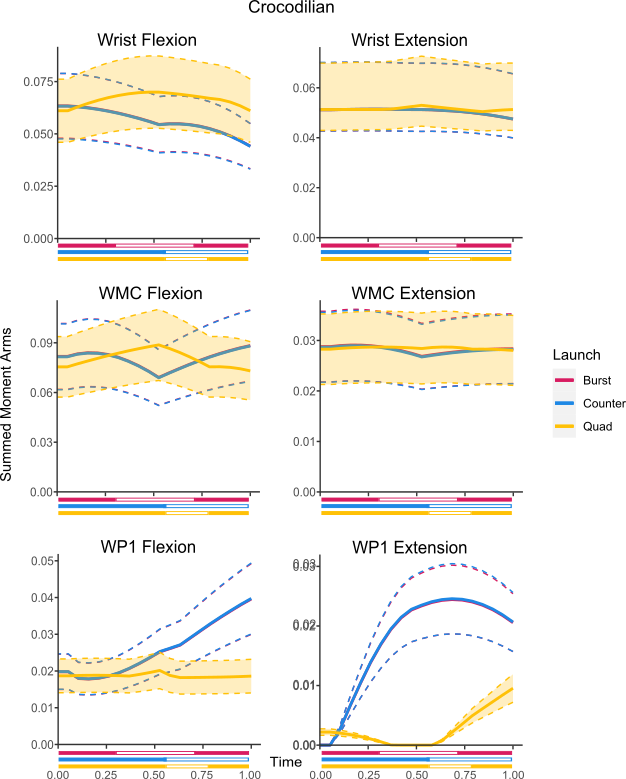


Figure S7: Summed moment arms in each hypothesised take-off motion for the hindlimb rotational DOFs utilising only muscles present in crocodilians. Solid lines indicate mean values following Monte Carlo simulation, dashed lines show estimated error, colouration indicates moment arm usage throughout the take-off.


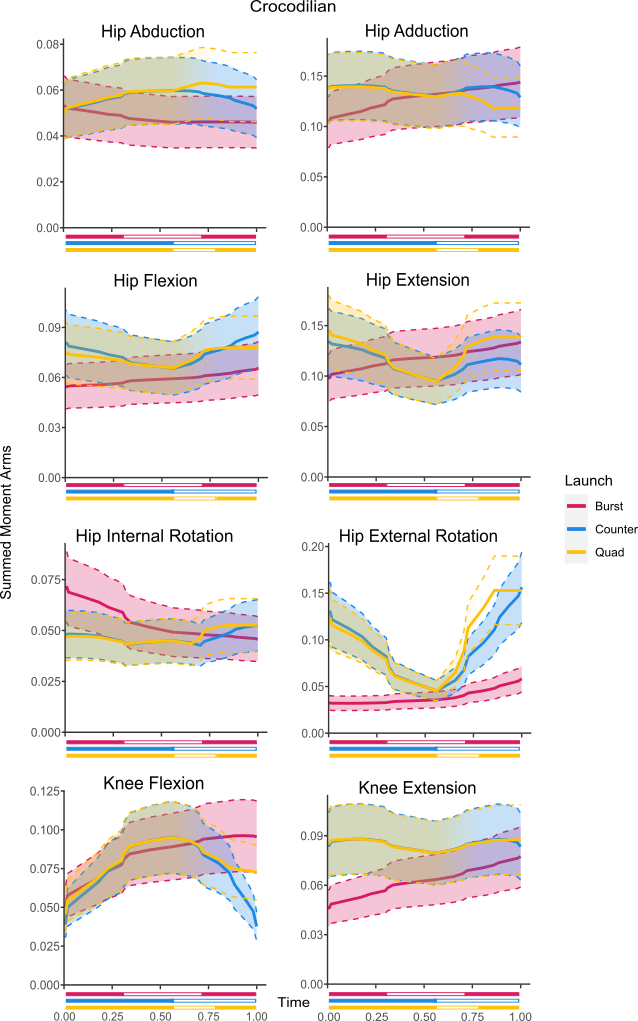


Figure S8: Colour Sensitive version of Figure 2. Standardised joint coordinate axis colours (RGB = XYZ) have been modified as follows: X axis has been changed to pink, Y axis has been changed to pale blue, and Z axis has been changed to yellow.


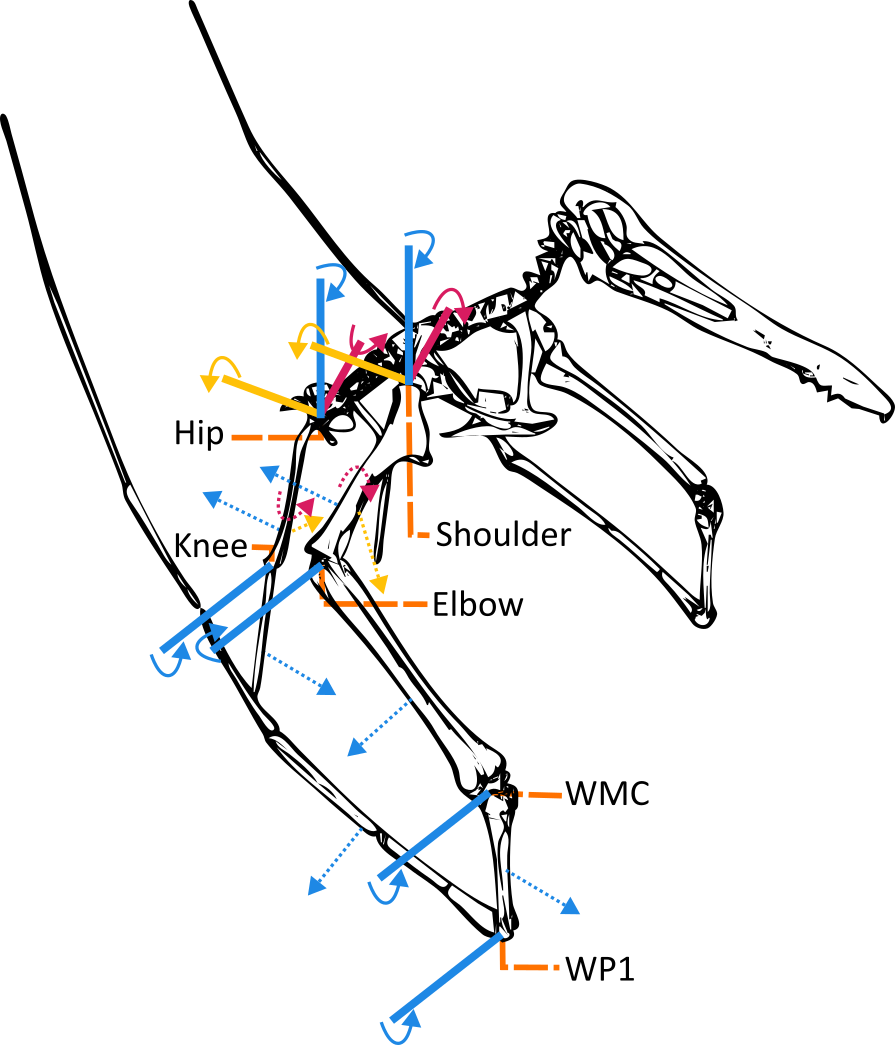

Supplement: Supplemental Information 4 [file peerj-12-17678-s004.docx]
